# A Case of Hemorrhagic Shock for a Ruptured Splenic Aneurysm Treated With REBOA-Assisted Surgery

**DOI:** 10.1155/cris/7264596

**Published:** 2025-07-09

**Authors:** Chiara D'Alterio, Cristina Carruezzo, Armando Raso, Arezia Di Martino, Roberto Santoro, Domenico Giannotti

**Affiliations:** ^1^Department of Surgery, Policlinico Umberto I, Sapienza University of Rome, Rome, Italy; ^2^Oncologic and General Surgery Unit, Ospedale Belcolle, Viterbo, Italy; ^3^Department of Radiology, Ospedale Belcolle, Viterbo, Italy; ^4^Diagnostic and Interventional Neuroradiology Unit, Azienda Ospedaliera di Rilievo Nazionale e di Alta Specializzazione Civico Di Cristina e Benfratelli, Palermo, Italy

**Keywords:** bleeding control, case report, noncompressible torso hemorrhage, nontraumatic hemorrhage, resuscitative endovascular balloon occlusion of the aorta (REBOA), splenic artery aneurysm

## Abstract

**Background:** Resuscitative endovascular balloon occlusion of the aorta (REBOA) is a technique aimed at temporarily interrupting or limiting blood flow through the aorta, which may be used as a bridge until definitive bleeding control by endovascular procedures or surgery. Despite the main current indication for its use is traumatic massive noncompressible torso hemorrhage, its application in end-stage nontraumatic abdominal and pelvic hemorrhage is progressively increasing.

**Case Presentation:** A 42 year-old male patient was brought to our hospital Emergency Department with acute onset of abdominal pain, hypotension, paleness, and diaphoresis. A computed tomography (CT) was performed evidencing a voluminous retroperitoneal hematoma caused by the rupture of an unknown splenic aneurysm. Emergency open splenectomy with resection of the splenic aneurysm and evacuation of the retroperitoneal hematoma was performed, with the assistance of the REBOA technique. The endovascular balloon was positioned in the aorta, proximally to the celiac axis (Zone 1), through a percutaneous femoral access by the interventional radiologist. Intermittent aortic occlusion enabled proximal bleeding control, adequate myocardial and cerebral perfusion, and allowed surgeons to safely and successfully perform splenectomy by resecting the splenic artery at the origin.

**Conclusion:** REBOA provides a rapid and minimally invasive hemodynamic control in severe hemorrhagic settings and its application in the initial management of nontraumatic abdominal hemorrhage should be strongly advised. Further studies with large sample size focusing on nontrauma patients are needed.

## 1. Introduction

**Figure 1:**
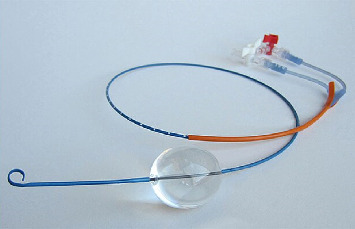
The REBOA catheter: the white sideport is labeled for balloon inflation, and the red is for arteriography or arterial pressure trasduction.

Resuscitative endovascular balloon occlusion of the aorta (REBOA) is a technique aimed at temporarily interrupting or limiting blood flow through the aorta. The use of an intra-aortic balloon was firstly described during the Korean conflict in 1954 by Hughes [1] who gave a presentation on traumatic hemorrhage control. REBOA consists in the introduction of an endovascular balloon catheter into the aorta through a small sheath in the femoral artery, which can be inflated at different levels above the estimated origin of the bleeding (Figure 1).

REBOA itself is not a solution for hemorrhage, but it may be used as a bridge that provides extra-time, while bleeding control is achieved by endovascular procedures or surgery. It enables to limit the hemorrhage and redistribute blood flow to the myocardium and brain, avoiding cardiovascular collapse and improving survival.

Although the concept of intra-aortic balloon occlusion is not new [2–4] REBOA has received a lot of attention in the last decade for its applicability in adult major trauma settings, and its use as a rescue device for managing life-threatening traumatic hemorrhage has increased dramatically [5–8]. Moreover, several recent studies have claimed that REBOA should be also indicated for the management of major hemorrhage of nontraumatic etiology [9–12].

The main indications can be divided into three groups: traumatic abdominopelvic hemorrhage, hemorrhage arising from ruptured abdominal aneurysms, and hemorrhage from other causes, such as postpartum bleeding, gastrointestinal bleeding or exsanguination during or after pelvic surgery [5].

Splanchnic aneurysms represent a rare and potential life-threatening condition. Splenic artery aneurysms (SAAs) are the most common type of splanchnic aneurysms, accounting for 60% of all cases, with an estimated prevalence of 1% in the population [13].

In this paper, we present the case of a 42 year-old male patient, who developed hemorrhagic shock following the rupture of an unknown splenic aneurysm and was successfully submitted to emergency open splenectomy, with the assistance of the REBOA technique. This case report has been reported in line with SCARE (Surgical CAse REport) criteria [14]. Written informed consent was obtained from the patient for the publication of this case report and accompanying images. Moreover, we reviewed the literature concerning the application of REBOA for end-stage nontraumatic abdominal and pelvic hemorrhage, and reported some considerations.

## 2. Case Presentation

**Figure 2:**
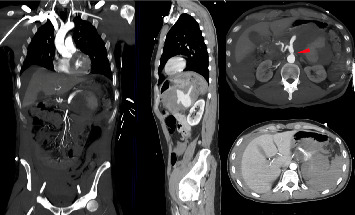
Contrast enhanced computed tomography scans. The red arrow indicates dissection of the celiac tripod at the origin.

In July 2022, a 42 year-old otherwise healthy male patient, positive to SARS-CoV2, was brought to our hospital Emergency Department with acute onset of abdominal pain, hypotension (110/60 mmHg), heart rate of 91 beats/min, paleness and diaphoresis, in absence of fever. The patient underwent central line placement and fluid resuscitation, blood tests, and contrast enhanced computed tomography (CT). The CT scan revealed a 20 cm × 15 cm blood-dense collection in the subdiaphragmatic area, located anteriorly to the left kidney and below the lower pole of the spleen (Figure 2). This collection was caused by a ruptured 2 cm aneurysm, situated at the middle of the splenic artery. The distal segment of the artery and the splenic hilum were not enhanced, being not visible. Associated to the aneurysm, the CT scan also highlighted dissection of the celiac tripod at the origin. The dissection flap extended to the splenic and the hepatic arteries. The CT revealed a large amount of blood effusion in the perihepatic and perisplenic areas, along the parietocolic channels down to the pelvis. During the CT exam, the patient became unstable (blood pressure, 70/40 mmHg; heart rate, 140 beats/min; respiratory rate, 45 breaths/min; and shock index, 2.0) and was urgently brought to the OR for surgical control of the bleeding.

Emergency open splenectomy with resection of the splenic aneurysm and evacuation of the retroperitoneal hematoma was performed, with the assistance of the REBOA technique. The surgical abdominal access was performed through a xipho-pubic laparotomy. The on-call interventional radiologists, who had been forewarned by the emergency physician, arrived when the patient was in the OR, bringing along the REBOA device. A femoral right retrograde access was obtained, despite the absence of a clearly palpable pulse due to the hypotension, a 7-Fr arterial sheath was positioned and the REBOA balloon was inserted. The balloon was positioned in the aorta, proximally to the celiac axis (Zone 1); since the maneuver could not be done under fluoroscopic guidance, the correct location was confirmed manually palpating the aorta and estimating the position of the device inside of it. Occlusion time of 10 min was alternated with deflation of the balloon, to guarantee blood flow to the abdominal organs and the lower limbs. When systolic blood pressure (SBP) dropped <60 mmHg during the deflation window, the balloon was inflated again. During occlusion time the SBP remained stable approximately around 110 mmHg. Every inflation/deflation maneuver was anticipated to the anesthesiologist to avoid sudden hypotension and cardiovascular arrest, and keep some hemodynamic stability. This resulted in a very tight teamwork among the interventional radiologist, the anesthesiologist and the surgeon.

**Figure 3:**
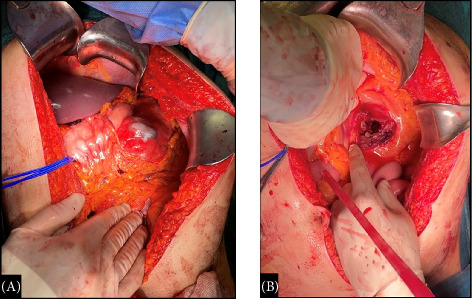
(A) Retroperitoneal pulsating mass of 20 cm diameter, which displaces the stomach anteriorly, the pancreatic body tail antero superiorly and the transverse mesocolon inferiorly. (B) Transverse mesocolon ripped in his inferior fold, with blood spillage.

Approximately, 2 L of blood and clots were drawn from the abdominal cavity. After the opening of the omental bursa, a 20 cm pulsating mass was found to displace the stomach anteriorly, the pancreatic body-tail antero superiorly and the transverse mesocolon inferiorly, the latter ripped in his inferior fold with blood spillage (Figure 3).

**Figure 4:**
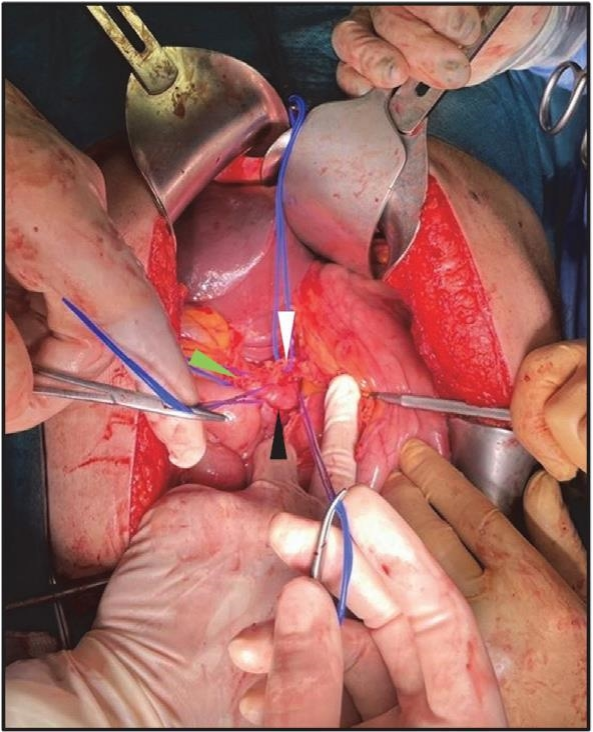
Preparation of the celiac tripod. Vascular structures are indicated by arrows: left gastric artery (white), hepatic artery (green), splenic artery (black). Splenic artery is isolated on a loop at its origin and on a distal loop.

The access to the celiac tripod and the isolation of its vascular structures was made difficult by the voluminous mass (Figure 4). The endovascular occlusion of the aorta allowed to avoid the obstacle and potential pitfalls associated with aortic cross-clamping, while reducing troublesome bleedings and maintaining hemodynamic stability. From laparotomy to definitive vascular control, a first cycle of 10-min inflation and 5-min deflation was performed, and during the second inflation period, the splenic artery was ligated at its origin from the celiac trunk. Therefore, the aortic occlusion was not needed anymore. Evacuation of the retroperitoneal hematoma and splenectomy were performed. Hemostatic matrix was applied and three 21-Fr peritoneal drainages were placed. Operative time was 2 h.

One unit of packed red blood cells (pRBCs) and four fresh frozen plasma packs were administered intraoperatively; other two units of pRBCs were administered postoperatively.

The patient remained in the intensive care unit for the first 24 h of recovery.

**Figure 5:**
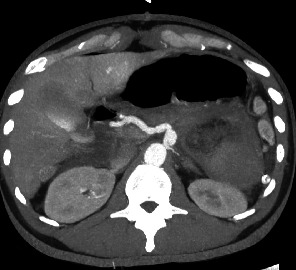
Control CT after 1 month.

The postoperative course was uneventful for surgical complications but was characterized by hypertensive crisis (up to 190/110 mmHg) treated with medical therapy. The patient was discharged after 14 days in a good state of health. The CT performed after 1 month showed no abdominal collections; the celiac trunk dissection remained unchanged (Figure 5). No complications occurred at 30- and 90-days and the patient was in good general condition at 1-year follow-up.

## 3. Discussion

REBOA is a technique aimed at temporarily interrupting or limiting blood flow through the aorta.

The aorta can be divided into three zones related to REBOA: Zone 1 extends from the origin of the left subclavian artery to the celiac artery; Zone 2 extends from the celiac artery to the lowest renal artery; and Zone 3 extends from the lowest renal artery to the aortic bifurcation.

Zone-1 inflation is indicated for imminent traumatic cardiac arrest for probable hemorrhagic cause and for abdominal and/or pelvic hemorrhage, while Zone-3 inflation is indicated for pelvic, junctional, or high extremity hemorrhage. Zone-2 inflation is usually not recommended due to the difficulty in occluding bleeding vessels at this location and the risk of visceral or renal vessels injury [15]. The balloon should be inflated until the blood pressure stabilizes or begins to rise and contralateral femoral pulse is stopped (8 mL for Zone 1 and 3 mL for Zone 3) [16].

Although the REBOA application in end-stage nontraumatic hemorrhage is progressively increasing, nontrauma patients still represent a low percentage of the population submitted to this procedure. In most studies, nontraumatic hemorrhagic patients are included and analised together with trauma patients [17–19].

However, clinical and physiopathological characteristics of traumatic and nontraumatic abdominal hemorrhage seem to be different [9]. Traumatic bleeding is typically greater in volume, caused by multiple bleeding sites and more likely to abrupt into the peritoneal cavity, while nontraumatic bleeding is more frequently slower in nature or limited into confined spaces. Bleeding in the nontrauma population can usually be supported with resuscitation and vasopressors, to a degree, before hemodynamic collapse [9]. Moreover, trauma and nontrauma populations often have different clinic characteristics. Traumatic cases are mostly represented by younger and previously healthy people, while nontraumatic hemorrhage usually involves older people suffering for several comorbidities [19].

The largest systematic review and meta-analysis, including nontrauma population, was published in 2018 and included 89 studies, with a total of 1482 patients submitted to the REBOA procedure for bleeding control. Fifty of the 89 studies focused on the application of REBOA in ruptured abdominal aortic aneurysm, and 21 on the REBOA use for other nontraumatic bleeding causes [5].

This review reported a 39.1% mortality in the population treated for ruptured abdominal aortic aneurysm, and a 3.1% mortality in other nontraumatic hemorrhagic patients. The outcomes of this study contributed to the development of the main clinical practice guidelines regarding the use of REBOA [10, 20, 21].

Splanchnic artery aneurysms represent a rare and potential life-threatening condition. In particular, SAAs, defined as an abnormal dilatation of the splenic artery, more than 1 cm in diameter, are the most common type of splanchnic aneurysms [22]. SAAs were first described in 1770 by Beaussier in autopsies [23], the first preoperative diagnosis was made in 1920 by Hoegler and the first surgical intervention was in 1940 [24]. SAAs mostly occur in the distal third of the artery (75%) followed by the middle third (20%) and are four times more common in females [25].

The etiopathogenesis is not fully understood but clear risk factors include female gender, history of multiple pregnancies due to hormonal and local hemodynamic events, fibromuscular dysplasia, defects of the tunica media, atherosclerosis, portal hypertension, and liver transplantation [25].

The majority of SAAs (80%) are asymptomatic and diagnosed incidentally during abdominal imaging [25]. Symptoms may be nonspecific and may include abdominal pain in the epigastrium and/or left upper quadrant, anorexia, nausea, and vomiting. Spontaneous rupture of the aneurysm is reported to occur in 2%–10% of all cases and is associated with a mortality rate that varies from 25% to 70% [26].

In case of rupture, the presentation can be a sudden onset of sharp abdominal pain and hypotensive shock. Sometimes it may present with the “double rupture phenomenon”, which was first described by Bockerman in 1930: the first hemorrhage is contained by the lesser omental sac, leading to a temporary tamponade, but after few hours the blood flows into the peritoneal cavity through the foramen of Winslow with resultant severe shock [22].

Our patient is a rare case in the literature, because he is a healthy young male with no trauma history and no risk factors, while most cases of splenic aneurysm rupture described in literature are reported in pregnant females or in patients with identifiable risk factors. This case represents our first experience with the REBOA technique.

Studies evaluating the use of REBOA in nontrauma or combined trauma/nontrauma settings reported higher rates of balloon-placement in the operative room (OR), rather than in the emergency department [9, 17]. It should be considered that performing REBOA in the emergency department may allow a more rapid temporary hemostasis, but it could potentially delay definitive hemostasis procedure if placed unnecessarily. OR intervention requires longer time to aortic occlusion and hemostasis, nevertheless it is not associated with increased in-hospital mortality. Therefore, emergency department REBOA should be indicated for patients who result nonresponders to initial resuscitative measures, while OR REBOA could be reserved for patients who respond to initial resuscitative measures in the emergency department and are promptly transferred to the OR [27].

Brenner et al. [17] reported 82% rate of OR REBOA among 11 patients submitted to REBOA procedure for nontraumatic hemorrhage, mostly for ruptured visceral aneurysms and massive upper gastrointestinal bleeding.

In our case, the operating room was already prepared, and the patient was immediately transferred there. One of the main advantages of REBOA is the ability to provide hemodynamic stabilization during laparotomy, with the option for rapid balloon inflation in the event of severe hypotension. Unfortunately, the REBOA device was not yet available when the patient arrived in the operating room. Therefore, we deemed it safer to proceed directly with surgery—considering the potential need for surgical aortic cross-clamping—rather than delay intervention while awaiting the device.

According to Brenner et al. [17] the benefit of performing the procedure with an open abdomen includes the ability to palpatorily confirm of the right location of the balloon, as well as to avoid potential pitfalls associated with aortic cross-clamping [9].

In our case, REBOA allowed a rapid and minimally invasive proximal control of the aorta. This enabled and limited the bleeding during the surgical procedure, thus improving visualization during medial spleen mobilization and proximal control of the splenic artery.

Complications of REBOA are numerous and can occur at all times of the procedure [16, 28]. Complications related to the arterial access include inability to obtain access, iatrogenic vessel injuries, such as dissection, rupture and perforation, leading to embolization, air embolisms, and peripheral ischemia. During the inflation of the balloon, the physician should be careful not to over-inflate, in order to avoid balloon rupture, or injury of the aorta. Referring to inflation/deflation time, radiologists and anesthesiologists have a fundamental role. In fact, time from inflation to deflation should be carefully measured. Given the tendency towards reperfusion injury, REBOA has a limited time window of application, before complications overcome benefits. Time from inflation to deflation should not exceed 30 min for abdominal hemorrhage and 60 min for pelvic hemorrhage [10]. Prolonged balloon inflation can lead to ischemia and potential cardiovascular complications after reperfusion, resulting in vasodilatation and refractory hypotension, not to mention multiple organ failure, including acute kidney injury, liver failure, spinal cord infarction, intestinal ischemia, and limb loss. Lastly, complications related to catheter removal include development of hematomas, pseudoaneurysms, thromboembolism, arterial dissection, and limb ischemia leading to amputation [16].

REBOA placement in the adequate zone requires high procedural competence and endovascular skills.

Partial REBOA technique was developed with the aim of reducing distal ischemia and extending the duration of REBOA use. This procedure consists in a slight deflation of the balloon, thus allowing a degree of flow beyond it. Several clinical and translational reports suggest that partial aortic flow restoration via partial aortic occlusion may mitigate the adverse effects of aortic occlusion on both proximal and distal vascular beds, while limiting ongoing bleeding [16].

Therefore, REBOA should be used only by people experienced in decision making surrounding its use and technically skilled in its application [29].

Procedural competence and short prehospital time are two determinant factors. Emergency physicians, acute care surgeons, anesthesiologists, interventional radiologists, critical care physicians and nurses must be familiar with REBOA and aware of its strengths, limitations, and potential complications before its use is adopted [30]. The low hospital distribution of this device could limit the learning curve of both operators and teams. In fact, it is demonstrated that survival is higher in centers with high vs. low REBOA-procedure volume [31].

## 4. Conclusion

Multidisciplinary approach should be the strategy of choice to improve the outcomes of hemodynamically unstable patients. REBOA provides a rapid and minimally-invasive hemodynamic control in severe hemorrhagic settings and its application in the initial management of nontraumatic bleeding should be strongly advised. However, further prospective studies with large sample size focusing on nontrauma patients are needed.

## Data Availability

Data sharing is not applicable to this article, as no new data were created or analyzed in this study.
